# Life after genetics

**DOI:** 10.1186/s13073-014-0086-2

**Published:** 2014-10-29

**Authors:** Jay Shendure

**Affiliations:** Department of Genome Sciences, University of Washington, Seattle, WA 98195 USA

## Abstract

Gene finding is a finite exercise, and a means to an end, rather than an end in itself. The field of human genetics should increasingly shift its attention from disease gene identification to following through on next steps, most importantly pursuing the biological mechanisms underlying genotype-phenotype associations.

## High-yield paradigms

We are in a period of rich discovery in human genetics and genomics. The ascertainment of genetic variation, previously the rate-limiting step for genetic analysis, has been revolutionized by new technologies for high-density genotyping, exome sequencing and genome sequencing.

Several high-yield paradigms - approaches that are exceptionally successful in generating discoveries - have emerged that exploit these technologies to uncover the genetic underpinnings of disease. For example, genome-wide association studies (GWAS), typically involving high-density genotyping in large case–control cohorts (effectively genome-wide scans because of linkage disequilibrium amongst common variants), have yielded thousands of reproducible genotype-phenotype associations [1]. Exome sequencing, by enabling the identification of highly penetrant rare variants and *de novo* mutations, is driving a renaissance in monogenic disease gene discovery, adding hundreds of new discoveries to the catalog of Mendelian disease genes [2]. Although most studies that have used exome sequencing to assess the contribution of rare variants to common diseases have been underpowered, a bright spot is the genetics of neuropsychiatric diseases, such as autism and intellectual disability, in which an excess of severe *de novo* mutations in probands highlights a clear path for pinpointing disease genes despite the extreme genetic heterogeneity of these phenotypes [3]. Analogous successes are taking place in cancer genetics, a field in which hundreds of genes are being newly implicated in disease by virtue of recurrent somatic mutations identified through exome or genome sequencing [4]. The distinctions between these high-yield paradigms are decreasing, as affordable whole-genome sequencing provides a comprehensive means for assessing the contribution of *de novo*, rare and common variations in both coding and non-coding regions of the genome to the full spectrum of human phenotypes.

## From genetics to variant interpretation and disease mechanism

Amid this success, it is important to remember that genetics is a means to one or several ends (such as a biological understanding of disease mechanisms, or identifying the basis of disease in a specific patient) rather than an end in itself. The ultimate impact of our field will depend not only on whether we can get the genetics right, but also on whether or not subsequent goals are achieved. At the same time, there are limits to what we can learn through genetic analysis alone. Following up on the fruits of human genetics will increasingly require that we experimentally characterize the variants and/or genes believed to underlie human phenotypes. There are at least four contexts in which this exhortation is relevant.

First, the linkage disequilibrium that underlies the efficiency of GWAS ironically limits their resolution. Although GWAS have been very successful in identifying reproducible ‘haplotype-phenotype’ associations (that is, multiple common variants in linkage disequilibrium with one another, all associated with the phenotype), the number of association signals for which the causal common variant(s) and/or genes are definitively known is disappointingly small. To the extent that the goal of GWAS is to identify dysregulated or dysfunctional genes (as opposed to loci) in common disease, GWAS are analogous to a compendium of promising but undeniably incomplete sentences. There are a few studies in which extensive experimental follow-up has identified the specific functional variants underlying an association, but these are few and far between, and the challenge of closing the gap between the number of genome-wide associations that are convincing and the number that are well understood is daunting.

Given the large number of genome-wide associations that require follow-up, we should be focused on defining generically applicable functional assays or workflows for chasing down causal variants within implicated haplotypes [5]. This may be facilitated by the fact that most causal variants underlying significant associations with common diseases are likely to be regulatory. For example, with the CRISPR/Cas9 system, it is possible to imagine systematically introducing candidate causal variants for a given association into a uniform genetic background in a relevant cell type and then measuring their impact (alone and in combination) on the transcriptional output of nearby genes. A separate challenge is that functional assays do not easily lend themselves to the consistent statistical standards that have been a mainstay of GWAS [6]. Therefore, an important question for the future is whether standards of experimental evidence for implicating specific variants or genes as ‘causal’ for associations can be defined and consistently applied.

Second, although we have been quite successful in identifying specific disease-associated genes in two domains - Mendelian disease (germline mutations) and cancer (somatic mutations) - we remain poorly equipped to interpret sequence variants that are observed in individual patients, that is, variants of uncertain significance (VUS). The underlying problem is unlikely to be solved by more sequencing: for example, the breast-cancer-associated genes *BRCA1* and *BRCA2* have been sequenced in over a million patients and yet a high proportion of returned results continue to be VUS. If anything, the challenge posed by VUS is likely to profoundly deepen as the clinical sequencing of human genomes accelerates and as the list of genes for which sequencing is clinically meaningful grows. In this context, functional assays may well be the way forward.

The functional assessment of clinically observed variants is nothing new. However, this has generally been retrospective: for instance, the functional characterization of alleles that have already been observed in one or more patients. The recent emergence of massively parallel approaches for dense mutagenesis and the functional analysis of specific sequences [7] may enable a different paradigm, in which all possible variants of a clinically relevant gene are functionally tested in advance of ever having been observed in a patient. Provided that the results of the functional assay correlate with clinical consequences, such ‘pre-computed’ interpretations could then be used in the very first instance in which the variant was observed in the clinic, thereby eliminating or minimizing VUS reports in that gene.

Third, the functional characterization of variants observed in patients can prove useful for the implication of a gene or locus in disease. However, it is important to be cautious about how such data are used and interpreted [6]. For example, it is often the case that a variant or mutation will highlight a reasonable candidate gene, but no ‘second family’ is available, motivating experimental characterization of the allele and/or gene to provide supporting evidence. But given the ‘narrative potential’ [8] of any given gene, as well as the non-trivial probability that a candidate mutation will be functional at the molecular level but not causal for the phenotype being investigated, the bar must be set extremely high for declaring success. It is far preferable that genes are implicated on the basis of genetic analysis alone, or that experimental information be used in a statistically rigorous fashion to boost discovery power in the first place; for example, to stratify genes [9] or variants [10] into subsets in which the strength of association correlates with molecular functionality.

Fourth, although genomics provides a systematic, genome-wide means of identifying a gene or genes in which variation contributes to the pathophysiology of a given disease, understanding the role of these gene(s) inevitably requires experiments. This is ostensibly a task for biologists rather than geneticists; however, geneticists bear some degree of responsibility for ensuring that the story does not end with genetics and, as such, there should be no barriers against geneticists delving deeply into the biology of gene mechanisms. Furthermore, the number of genes implicated by genetic approaches in human phenotypes but whose biological function remains poorly understood is easily in the thousands. The armamentarium of genomic approaches for observational (for example, transcriptional profiling) and perturbational (for example, genome-wide knockdown or knockout screens) experiments may represent useful approaches for advancing our fundamental understanding of the biological role(s) of implicated genes in a scalable fashion.

In summary, to shed further light on the plethora of established gene discoveries and locus associations, the onus is on geneticists to take the next steps. A strength of forward genetic approaches for gene finding has been that they are systematic or ‘hypothesis free’; that is, all genes are *a priori* equally likely candidates at the outset of a study. This principle has served our field enormously well, as it provides the freedom to make discoveries in expected corners [11]. Although particular genes and variants will of course require systems of experimental analysis that are specific to the contexts and manner in which they function, it may nonetheless prove powerful to carry this general philosophy forward where possible; for example, genome-wide screens for genetic or physical interactions, building distributions of variant effect sizes, and so on.

A foundational goal of human genetics may be to unravel the genetic basis of human disease, but the ultimate impact of our field will be measured by whether and how this knowledge is put to use. Furthermore, gene finding is a finite exercise, or at least subject to the law of diminishing returns. Although the day when the apples get too high to reach may still be in the future, we should not lose sight of the fact that the ground is already littered with apples. We must get going on carrying these discoveries forward, lest we get buried in our own success.

## Abbreviations

GWAS: Genome-wide association studies
VUS: Variants of uncertain significance
